# The Relative Efficiency of Medicaments for the Sterilization of Tooth Structure

**Published:** 1918-08

**Authors:** M. M. Brooks, W. A. Price


### THE RELATIVE EFFICIENCY OF MEDICAMENTS FOR THE STERILIZATION OF TOOTH STRUCTURE

BY M. M. BROOKS AND W. A. PRICE

This paper is limited to the sterilization of infected dentin and cementum as involved in root canal infection following the death of the dental pulp. A very interesting historical survey serves to orient the reader in the problem, and furnishes a valuable bibliography.

The authors recognize that the following structures and conditions exist as important factors: (1) The root canal to which the infection has access, and through which the infection can reach the dentin; (2) the dentin with its tubuli; (3) through the foramen or foramina the infection can reach the cementum, which probably can also be infected from the dentin; (4) the periapical tissues, which constitute not only the pabulum but also a locked area which may contain an infected culture medium, which may reinfect the cementum and dentin and the root canal contents.

The experiments have been arranged in the following order: (1) A study of the ability of the medicaments to sterilize dentin and cementum under the most ideal conditions, irrespective of compatibility with clinical conditions; (2) the study of the ability of the medicaments to even retain the sterility of a medicated dressing when placed in an infected root canal without the influence of a periapical infection; (3) the influence of a periapical infection upon these last-named conditions; (4) the ability of medicaments placed within the root canal to sterilize infected dentin and cementum while these tissues are surrounded by a periapical infection, including a study of the areas of these structures that are most readily sterilized; (5) a study of the time factor, and (6) a study of silver-ammonium-formalin and Dakin solutions.

The results of the first series of experiments show that even under ideal conditions, where the medicament bathes both the inner and the outer surfaces of the infected tooth structure, and even where the mass of the medicament is relatively large and without compatibility for the surrounding tissues, the dentin and cementum were not disinfected except with a few medicaments, namely, formalin in all strengths (in the protocols this is given in percentages of formaldehyde—40, 30, 20, 10, 5, and 1) and chlorphenol. The great majority of results were negative and in direct contradiction to the general teachings and expectations in current dental practice. It is very difficult to destroy bacteria growing in dentin and cementum.

The conclusions to the second series of experiments are that the most radical disinfectant is dichloramin-T. This medicament in a concentration of fifteen per cent. as used in these experiments gave one hundred per cent. efficiency in all treatments, but also produced irritation of living tissue. A further observation on the lowest percentage needed for root canal sterilization and the amount of time for the production of this condition will be continued. This drug may, in dilute solution of one per cent. or less, be both safe and efficient, but it is one of the most painful and destructive medicaments tried in practical cases. The members of the profession are warned never to place the fifteen per cent. or even five per cent. solution of dichloramin-T in eucalyptol, its solvent, in any tooth or on tissues. (However, the newer solvent, chlorcosane, is devoid of this irritative effect.)

The third series of experiments shows that under the conditions specified above, the medicaments used—Buckley's phenol compound, dichloramin-T fifteen per cent., formocresol, iodin-creosote, iodin U. S. P., formalin U. S. P., phenol U. S. P., and oil of cloves fifty per cent.—are not ideal disinfectants. The interpretation of this experiment is probably that the penetration of the disinfectant is not sufficient to reach the remote and minute rescesses of the tubuli. To find some disinfectant or method that will meet this requirement is the subject of the remaining experiments, although these are not yet completed.

The fourth series emphasizes that the efficiency of a medicament to sterilize *all* tooth structures should be the criterion of a disinfectant.

Experiments upon Howe's silver nitrate-ammonia-formalin method (see *Dental Cosmos* for September, 1917), incline the authors to believe that a perfected technic will produce sterilization of the tooth structures which are reached by the medicament in so short a time as ten minutes.

The experiments on Dakin's chlorazene and dichloramin-T give much encouragement for their use. They are, however, painful and destructive in over five-tenths per cent. strength. It must be remembered that this conclusion was drawn when eucalyptol was the solvent; the solvent chlorcosane eliminates this irritative action, so that percentages of dichloramin-T of over six per cent. may be used.

Among the general summary the points which are of special interest are: (1) The efficiency of a root treatment is greater a few hours after it has been placed in the tooth than after several days' or even one day's time; (2) all areas of cementum and dentin are difficult to sterilize, as well as tend to reinfection when the medicated root dressing is left more than a few hours; (3) the medicaments that are most efficient, namely, silver nitrate and formalin, are very objectionable, the former by its discoloration and the latter by its destructive and irritating properties, except when used very dilute and for a short time, and then quite efficiently. The signification of this last cryptic phrase is revealed in *Oral Health* for April, 1918, by Price, in a description of the method of Cameron of Girard College, Philadelphia, for the sterilization of root canals, which Price characterizes as superior in results to any other current method. Put four per cent. solution of formalin into the accumulated debris and use it as a solvent for washing out the pulp chamber and canals, but not undertaking to put any of it through or to the apex. Follow this with a two per cent. solution of formalin. The canals are not flooded, but merely moistened with this solution. Place a hot wire into the canals, and when the solution is raised in temperature to about 120 degrees, which will not be particularly unpleasant to the patient, formaldehyde gas is given off rapidly and profusely, and passes into the structures of the tooth. Teeth treated in this way show a very high percentage of efficiency, and the laboratory test of Price and collaborators have confirmed it as more efficient than the methods used in the past. All is done at one short sitting, and the canals are immediately filled. In this way the irritative after-effect of formalin is quite obviated.—Extracted by *Dental Cosmos* from *Journal of National Dental Association.*

